# Fast in vitro methods to determine the speed of action and the stage-specificity of anti-malarials in Plasmodium falciparum

**DOI:** 10.1186/1475-2875-12-424

**Published:** 2013-11-16

**Authors:** Claire Le Manach, Christian Scheurer, Sibylle Sax, Sarah Schleiferböck, Diego Gonzalez Cabrera, Yassir Younis, Tanya Paquet, Leslie Street, Peter Smith, Xavier C Ding, David Waterson, Michael J Witty, Didier Leroy, Kelly Chibale, Sergio Wittlin

**Affiliations:** 1 Department of Chemistry, University of Cape Town, Rondebosch, 7701, South Africa; 2 Parasite Chemotherapy Unit, Swiss Tropical and Public Health Institute, Basel, Switzerland; 3 University of Basel, Basel CH-4003, Switzerland; 4 Division of Clinical Pharmacology, Department of Medicine, University of Cape Town, Observatory 7925, South Africa; 5 Medicines for Malaria Venture, ICC, 20 Route de Pré-Bois, PO Box 1826, Geneva 1215, Switzerland

**Keywords:** *Plasmodium falciparum*, Speed of action, Stage-specificity

## Abstract

**Background:**

Recent whole cell in vitro screening campaigns identified thousands of compounds that are active against asexual blood stages of Plasmodium falciparum at submicromolar concentrations. These hits have been made available to the public, providing many novel chemical starting points for anti-malarial drug discovery programmes. Knowing which of these hits are fast-acting compounds is of great interest. Firstly, a fast action will ensure rapid relief of symptoms for the patient. Secondly, by rapidly reducing the parasitaemia, this could minimize the occurrence of mutations leading to new drug resistance mechanisms.

An in vitro assay that provides information about the speed of action of test compounds has been developed by researchers at GlaxoSmithKline (GSK) in Spain. This assay also provides an in vitro measure for the ratio between parasitaemia at the onset of drug treatment and after one intra-erythrocytic cycle (parasite reduction ratio, PRR). Both parameters are needed to determine in vitro killing rates of anti-malarial compounds. A drawback of the killing rate assay is that it takes a month to obtain first results.

**Methods:**

The approach described in the present study is focused only on the speed of action of anti-malarials. This has the advantage that initial results can be achieved within 4–7 working days, which helps to distinguish between fast and slow-acting compounds relatively quickly. It is expected that this new assay can be used as a filter in the early drug discovery phase, which will reduce the number of compounds progressing to secondary, more time-consuming assays like the killing rate assay.

**Results:**

The speed of action of a selection of seven anti-malarial compounds was measured with two independent experimental procedures using modifications of the standard [^3^H]hypoxanthine incorporation assay. Depending on the outcome of both assays, the tested compounds were classified as either fast or non-fast-acting.

**Conclusion:**

The results obtained for the anti-malarials chloroquine, artesunate, atovaquone, and pyrimethamine are consistent with previous observations, suggesting the methodology is a valid way to rapidly identify fast-acting anti-malarial compounds. Another advantage of the approach is its ability to discriminate between static or cidal compound effects.

## Background

Malaria is one of the most devastating infectious diseases in the world and is responsible for an estimated 544,700-904,000 deaths each year [1]. It annually affects hundreds of millions of people, principally in sub-Saharan Africa, Asia and South America, with young children and pregnant women being particularly at risk. The fight against malaria remains a constant challenge as parasites manage to adapt and develop resistance mechanisms, making them less sensitive to the latest drugs. A critical example of the latter is the observed delay in parasite clearance time following arteminisin-based combination therapy (ACT) in Southeast Asia [2,3]. Since ACT has been adopted worldwide as first-line treatment, the rise of resistance to these drugs could lead to a malaria resurgence. The development of new anti-malarial agents is thus urgently needed to counter the spread of drug-resistant malaria.

The last few years have seen the development of innovative high-throughput screening that allowed testing of millions of compounds from diverse libraries against whole parasites [4]. GlaxoSmithKline [5] (GSK), Novartis [6] and St Jude’s [7] have screened their collections against the erythrocytic stages of *Plasmodium falciparum.* More than 20,000 hits that inhibit or kill the parasite at a concentration of less than 1 μM were identified. As a result, an explosion of numbers of active new chemotypes that can potentially be developed as anti-malarial drugs have been reported [8-10].

However, one of the main current challenges is to be able to assess the potential of these chemotypes early in the drug discovery process. New drugs should ideally have a rapid onset of action to relieve patient symptoms as fast as possible and so that a minimal number of parasites survive after exposure to the drug, thereby minimizing the resistance selection risk [9-11]. In this prospect, researchers at GSK in Spain have developed a killing rate assay that allows measuring the effect of a compound on parasite viability over time by determining its killing rate and speed of action [12]. A drawback of this method is that first results cannot be expected before four weeks.

In order to get a quicker evaluation of the speed of action of a compound and to solve the lack of filters in the early stage of the drug discovery testing cascade, a method based on modifications to the standard [^3^H]hypoxanthine incorporation assay was developed. The first results were achieved within a week.

**Figure 1:**
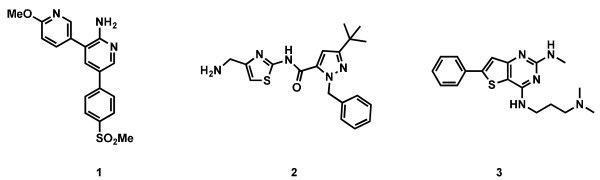
Structures of compounds 1, 2 and 3.

The method was validated with the anti-malarial standards chloroquine, artesunate, atovaquone, and pyrimethamine and was also used to determine the speed of action of three novel compounds (**1**[13], **2**[14] and **3**) (Figure 1), derived from different series identified during screening of Biofocus libraries [15].

## Methods

### Chemicals and materials

Chloroquine (MW: 516), artesunate (MW: 384), atovaquone (MW: 367) and pyrimethamine (MW: 249) were obtained from Sigma Aldrich (Switzerland).

**Figure 2:**
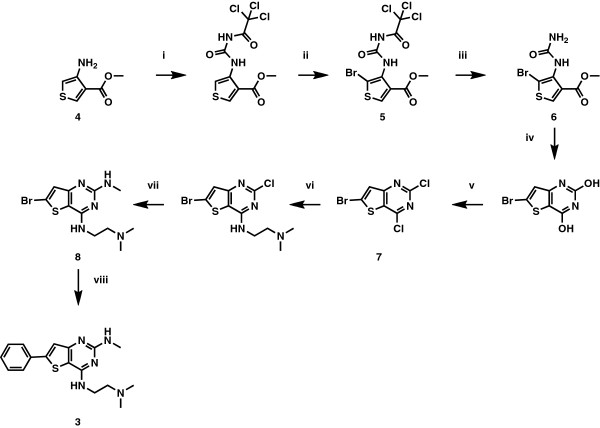
**Synthetic route for compound 3.** Reagents and Conditions: (i) trichloroacetyl isocyanate (1 eq), THF, 0°C to r.t, 2 h, 91%; (ii) bromine (3.8 eq), acetic acid, 0°C to 80°C, 14 h, 65%; (iii) ammonia, CH_3_OH, 0°C to r.t., 30 min, 76%; (iv) *t*-BuOK, DMF, RT, 14 h, 99%; (v) POCl_3,_*N,N-*dimethyl aniline (0.5 eq), 130°C, 14 h 88%; (vi) 3-dimethylaminopropylamine (1 eq), Na_2_CO_3_ (2 eq), EtOH, r.t., 14 h, 72%; (vii) 2 M methyl amine in THF (10 eq), dioxane, sealed tube, 100°C, 14 h, 83%; (viii) phenylboronic acid (1.1 eq), Pd(PPh_3_)_2_Cl_2_ (0.05 eq), aq. 1 M K_2_CO_3_ (1.05 eq), DMF, 90°C, 14 h, 63%.

Compounds **1** and **2** were synthesized using the experimental procedures previously described [13,14]. Compound **3** was obtained from a 7-step synthesis from commercially available reagent **4** (Figure 2). Reaction of **4** with trichloroacetyl isocyanate in THF, followed by bromination gave compound **5** in 91% yield. Subsequent treatment with ammonia in methanol afforded intermediate **6**, which cyclized under basic conditions. Subsequent chlorination with POCl_3_ gave key dichloro intermediate **7**. Two consecutive N-substitution reactions with 3-dimethylaminopropylamine under basic conditions and methyl amine respectively gave intermediate **8**, which underwent a final Suzuki cross-coupling reaction with phenylboronic acid to give the desired compound **3** as a white solid (Gonzalez Cabrera D *et al.*: 2,4-Diamino-thienopyrimidines as orally active antimalarial agents. Manuscript submitted). All three compounds were analysed by HPLC prior to biological experiments and were found to be >98% pure.

[^3^H]hypoxanthine was purchased from ANAWA Trading SA (Wangen, Switzerland). Anti-malarial compounds were dissolved in DMSO at 10 mg/mL. The stock solutions were kept at 4°C for not more than six months. Dilutions were prepared from stock solution immediately before use. The DMSO concentration (<0.5%) in the experiments had no inhibitory effect on parasite cultures.

### Parasite cultivation

The drug-sensitive *Plasmodium falciparum* strain NF54 (airport strain from the Netherlands) was provided by F Hoffmann-La Roche Ltd (Basel, Switzerland). The parasites were cultivated at 37°C as has been described [16]. Briefly, the medium consisted of RPMI 1640 supplemented with 0.5% ALBUMAX II, 25 mM Hepes, 25 mM NaHCO3 (pH 7.3), 0.36 mM hypoxanthine, and 100 g/ml neomycin. Human erythrocytes served as host cells. Cultures were maintained at 37°C in an atmosphere of 3% O_2_, 4% CO_2_, and 93% N_2_ in humidified modular chambers.

### IC_50_ speed assay

**Figure 3:**
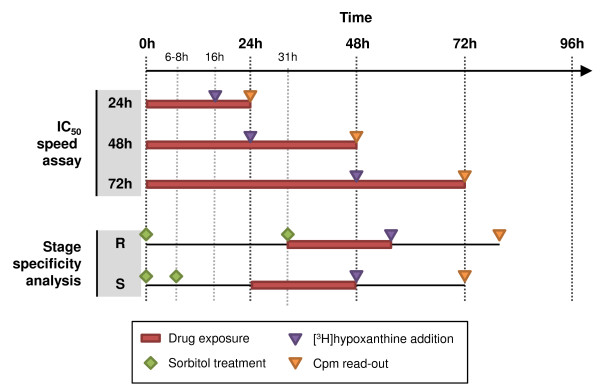
**Schematic representation of the two*in vitro*assays.** The “IC_50_ speed assay” and “stage-specificity analysis” are performed with unsynchronized and synchronized parasite cultures, respectively. R, rings; S, schizonts.

A schematic representation of the IC_50_ speed assay is shown in Figure 3. Briefly, parasite growth in the presence of anti-malarial compounds was assessed using the [^3^H]hypoxanthine incorporation assay and expressed as IC_50_ values [17]. For each compound, three incubation times were employed: 72 (standard assay time), 48 and 24 hours. In the case of the 72- and 48-hour assays, radioactive hypoxanthine was added for the last 24 hours. In the case of the 24-hour assay, [^3^H]hypoxanthine was added during the last eight hours. IC_50_ values in the standard 72-hour assay for chloroquine, artesunate, atovaquone, pyrimethamine, **1**, **2** and **3** were previously found to be 5.1 ± 0.8 [14], 1.6 ± 0.1 [14], 0.38 ± 0.04 [18], 5.6 ± 0.5 [18], 18 ± 1 [13], 26 ± 4 [14] and 9.5 ± 2.6 [16] ng/mL.

### Stage-specificity analysis

Using synchronized cultures of NF54, the concentration-dependent growth of ring and schizont forms in the presence of anti-malarial compounds was measured as previously described [18].

As depicted in Figure 3, NF54 cultures were synchronized twice with 5% D-sorbitol. To obtain early schizont stages, the second sorbitol treatment was done six to eight hours after the first. This procedure provided initially a parasite culture containing ≥80% young trophozoites (up to 20 hours old), which after cultivation of another 16 hours resulted in early schizont stages (up to 36 hours old).

To obtain ring forms, the second sorbitol treatment was performed 31 hours after the first, yielding a parasite culture with ≥80% rings (up to three hours old).

One 96-well microtitre plate for each of the two synchronous stages was then incubated for 24 hours with two-fold serial dilutions of anti-malarial compounds. Investigated concentrations ranged from 1.6-100 × the previously determined IC_50_ of each compound in a standard 72-hour assay. Following incubation, the plates were washed 4x resulting in a >1,000-fold dilution of free compound followed by another incubation period of 24 hours at 37°C in the presence of [^3^H]hypoxanthine. The plates were then frozen at -20°C or directly processed as described [17].

## Results

The herein described methodology consists of two independent experimental approaches. The first assay was named “IC_50_ speed assay” and is performed with unsynchronized cultures, and the second one “stage specificity analysis” (Figure 3).

**Figure 4:**
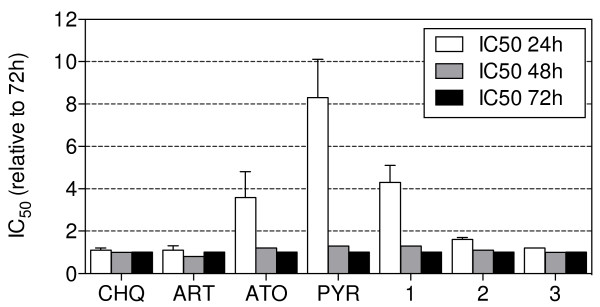
**IC**_**50**_**speed assay (NF54 unsynchronized culture; mean ± SD of n = ≥3 independent assays).** CHQ, chloroquine; ART, artemisinin; ATO, atovaquone; PYR, pyrimethamine.

In the IC_50_ speed assay, IC_50_ values were determined side-by-side for the four anti-malarial standards chloroquine, artesunate, atovaquone, and pyrimethamine as well as the three novel compounds **1**, **2** and **3** (Figure 1) after total incubation times of unsynchronized parasite cultures for 24, 48 and 72 hours (Figure 4, Additional file 1: Table S1). The 24-hours assay with chloroquine, artesunate, **2** or **3** resulted in very similar IC_50_ values compared to the standard 72-hour assay (ratio of IC_50_ 24 hours/IC_50_ 72 hours was 1.1, 1.1, 1.6 and 1.2). The IC_50_s of atovaquone, pyrimethamine and **1** were 3.6-, 8.3- and 4.3-fold higher at the 24-hour time point compared to the those generated at the 72-hour time point (Figure 4, Additional file 1: Table S1). These data, obtained after three working days, constituted the first indication that the latter compounds were not fast-acting molecules (Table 1).

**Table 1:** Data overview of IC50 speed assay and stage specificity analysis

**Compound**	**Assay 1 (A1):**	**Conclusion A1**	**Assay 2 (A2):**	**Conclusion A2**	**Concluded speed of action**
	IC50 speed assay		Stage specificity analysis		
	(ratio of IC50 24 h/IC50 72 h)^a^		(at 6-100x IC50)		
Chloroquine	1.1	Fast	>95% action on R^b^	Fast (cidal)	**Fast (cidal)**
			>95% action on S^b^		
Artesunate	1.1	Fast	>95% action on R	Fast (cidal)	**Fast (cidal)**
			>95% action on S		
Atovaquone	3.6	Not fast	~50-70% action on R	Not fast (cidal)	**Not fast (cidal)**
			~75-90% action on S		
Pyrimethamine	8.3	Not fast	~5% action on R	Not fast (cidal)	**Not fast (cidal)**
			~90% action on S		
1	4.3	Not fast	~0-75% action on R	Not fast (cidal)	**Not fast (cidal)**
			>95% action on S		
2	1.6	Fast/Not fast	~40- >95% action on R	Not fast (cidal)	**Not fast**
			>95% action on S		
3	1.2	Fast	>95% action on R	Fast (cidal)	**Fast (cidal)**
			>95% action on S		

^a^Ratios are means from ≥ 3 independent experiments performed with P. falciparum strain NF54.

^b^R and S are abbreviations for the ring and schizont stages, respectively.

The stage-specificity assay was performed with either young rings or young schizonts, which were incubated for 24 hours with serial dilutions (concentrations ranging from 1.6-100× of the previously determined IC_50_ values) of the above-mentioned seven anti-malarial compounds. Subsequently free compounds were removed and plates again incubated in the presence of radioactive hypoxanthine for 24 hours. Initial results from this test can be obtained within four working days.

**Figure 5:**
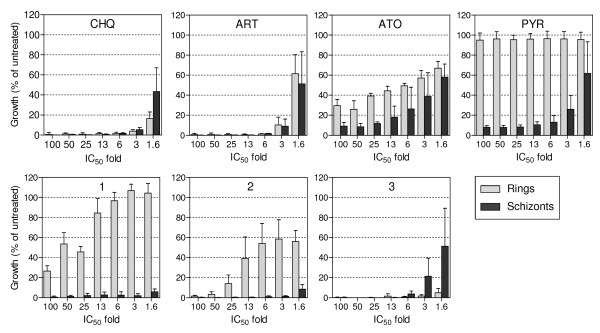
**Stage-dependent effects of chloroquine, artesunate, atovaquone, pyrimethamine and compounds 1, 2 and 3 on synchronous cultures of*Plasmodium falciparum*strain NF54.** Cultures were exposed to 7 different concentrations of the compound for 24 h. After removal of the compounds, parasites were incubated for another 24 h in the presence of [^3^H]hypoxanthine. Compound effects are expressed as the percentage of growth of the respective development stage relative to an untreated control. The open bar is the ring stage and the filled bar is the schizont stage. Each bar represents the mean ± SD of n = ≥3 independent experiments. CHQ, chloroquine; ART, artemisinin; ATO, atovaquone; PYR, pyrimethamine.

The rationale to perform the stage-specificity assay was to challenge the data from the IC_50_ speed assay. Assuming, for instance, that the IC_50_ speed assay would categorize a compound as non-fast-acting, and the stage-specificity assay would indicate a preferred action on young schizonts, then the latter data could provide an explanation as to why a compound is acting slowly. A comparable scenario turned out to be the case for pyrimethamine. The above mentioned 8.3-fold IC_50_ 24 hour/IC_50_ 72 hour shift in the IC_50_ speed assay (Figure 4) could be explained by the minor activity against rings (Figure 5). The observation that pyrimethamine acts only on older forms (Figure 5) is not unexpected, since similar data have been published previously [19].

Atovaquone data from the IC_50_ speed assay suggests that this drug has a slow action. This was also supported by the data from the stage-specificity assay, showing not more than 70 and 90% action against rings and schizonts (Figure 5). Those observations are in line with recent reports from 2 groups [20,21], who indicate that atovaquone has weak inhibitory activity against the rings and schizonts for the lines tested.

It is likely that the inhibitory effect of compounds acting even slower than atovaquone or pyrimethamine would be poorly represented in the here described methodology, since the maximum drug incubation time is 72 h. For instance in the case of azithromycin, a drug with a so-called delayed death phenotype above 72 h, it has been described previously that significant parasite growth reduction can only be observed by extending the drug exposure time to at least 96 h [12,22]. The prolongation of the total incubation times of the here described assays should help to address this fact.

Chloroquine and artesunate were found to be fast-acting compounds (Figure 4) and showed similar activity against rings and schizonts (Figure 5). These observations fit with what is known from the literature about those compounds [12,18,23].

Similarly, in the case of two of the three novel compounds, there was a good match between the two assays. Compound **1** showed a similar stage-specific profile like pyrimethamine, with predominantly strong action against young schizonts. The activity against young ring forms was <20% at concentrations up to 13× the IC_50_ and only ~60-75% in the higher concentration range. Compound **3** showed high activity against both blood stages (>95% on rings and schizonts in the 6-100× IC_50_ range), which suggests it is a fast-acting compound, similarly to what the IC_50_ speed assay had already demonstrated.

The only molecule showing contradictory results between the assays was compound **2**. The data from the IC_50_ speed assay suggests a relatively fast action (1.6-fold IC_50_ 24 hour/IC_50_ 72 hour shift). However, the stage-specificity assay proposes a slow action on rings (~40-60% activity at concentrations up to 13× the IC50 and >95% activity only at the two highest concentrations). Theoretically it should indeed be possible to see different outcomes, e g, it can be expected that due to the constant presence of compound during the assay incubation time of the IC_50_ speed assay, the latter would likely not be the right assay to detect if a compound is acting in a static or a cidal manner, because a viable but metabolically inactive parasite would be measured as dead. The stage-specificity assay, however, should have the potential to discriminate between static and cidal compounds, because of the washing procedure implemented after the compound incubation period. The washing is expected to remove the compound during the time when the metabolic activity is being determined. Since the data from both assays were in agreement in the case of all compounds except for molecule **2**, it can be expected that they should have cidal activities (Table 1).

The lack of correlation between the two assays in the case of compound **2** suggests that performing only one of them might not be acceptable. An exception could be anti-malarial compounds with certain defined phenotypes. There the assays could be interchangeable. However, in the absence of such knowledge, and until the assays are further validated with compounds of more chemical diversity, we do not recommend this approach.

## Conclusions

The results obtained for the anti-malarials chloroquine, artesunate, atovaquone, and pyrimethamine are consistent with previous observations [12,18-21,23]. This suggests that the assays described here are valid to rapidly discriminate between fast- and slow-acting anti-malarial compounds, providing valuable information to guide and accelerate the development of new classes of anti-malarial compounds.

## Abbreviations

PRR: Parasite reduction ratio; ACT: Arteminisin-based combination therapy; CHQ: Chloroquine; ART: Artemisinin; ATO: Atovaquone; PYR: Pyrimethamine.

## Competing interests

The authors declare that they have no competing interests.

## Authors’ contributions

CLM, CSC, XDI, DLE and SWI designed research. CSC, SSA and SSC performed research. All authors analysed data. All authors from UCT contributed to reagents. CLM and SW wrote the manuscript. All authors had full access to all data in the study and read and approved the final manuscript.

## Supplementary Material

Additional file 1: Table S1 IC50s (ng/ml) of all speed assays (NF54 unsynchronized culture). Click here for file
